# Cultivating a psychological health and safety culture for interprofessional primary care teams through a co-created evidence-informed toolkit

**DOI:** 10.1177/08404704241263918

**Published:** 2024-07-23

**Authors:** Jelena Atanackovic, Melissa Corrente, Sophia Myles, Houssem Eddine Ben-Ahmed, Karina Urdaneta, Kamlesh Tello, Magdalena Baczkowska, Ivy L. Bourgeault

**Affiliations:** 1 6363 Canadian Health Workforce Network, Ottawa, Ontario, Canada.; 2 434957 Mental Health Commission of Canada, Ottawa, Ontario, Canada.

## Abstract

The psychological health and safety of healthcare workers workplaces and learning environments impacts the quality of healthcare services. To facilitate the psychological health and safety of interprofessional primary care teams, we curated a bilingual toolkit of 122 psychological health and safety resources comprising a multi-level categorization addressing individual, team, organization, and system-level interventions. The resources in the toolkit are organized by 7 themes, based on a clustering of the 15 psychosocial factors. Adopting the framework built on the 7 themes, this article describes the toolkit development process and how it addresses the key factors for psychologically healthy and safe workplaces to foster interprofessional collaboration. Implementation of the interventions in the toolkit is an important next step for which health system leadership is critical. Additionally, we identify several gaps and call on researchers, educators, and health leaders to address them in their future work.

## Introduction

Psychological Health and Safety (PH&S) is embedded in the a way people interact with one another as well as the way working conditions and management practices are structured within the workplace.^1^ A psychologically healthy and safe workplace is an environment where “any member of the team feels comfortable: to ask questions without fear of being foolish; is able to acknowledge limitations without fear of being labelled incompetent; and can make suggestions without fear of being labelled disruptive.”^2^ Some data show a limited attention devoted to PH&S in the workplace. For instance, a study by the Mental Health Commission of Canada (MHCC) reported that 70% of employees are concerned about the PH&S of their workplace, and 14% do not think their workplace is healthy or safe at all.^3^ This is especially problematic in healthcare settings, given that the PH&S of healthcare workers leads not only to improved team learning and performance but also to continuous quality improvement environments affecting patient care.^4^ An effective PH&S strategy is beneficial to employers, impacting workforce stability, productivity, insurance costs, risk of legal or regulatory sanctions, and even their financial situation.^5^ It is also advantageous for employees, significantly impacting their health, morale, work-life balance, and ability to perform at their highest capacity.^5^

Over the past few decades, the provision of primary care in Canada has shifted towards interprofessional team-based models.^6,7^ Interprofessional Primary Care (IPC) teams involve two or more healthcare providers coordinating to ensure care for a patient.^7^ While some provinces have transitioned to primary care teams more than two decades ago, others such as British Colombia have adopted these changes more recently.^8^ Despite the differences in the structure and composition of primary care teams between different provinces, the main feature of an IPC model is “care that is coordinated and integrated within the team and across the health system as a whole.”^7^

As primary care systems across Canada evolve toward team-based approaches,^6,9^ it is critical to ensure that teams and training programs have the support they need to establish and work in a psychologically healthy and safe environment. To that end, our project aimed to develop and a bilingual “self-serve” platform of PH&S resources to support teams and training programs. More specifically, we developed and curated the Psychological Health and Safety Toolkit for Primary Care Teams and Training Programs, a bilingual toolkit with over 120 resources. The toolkit comprises a multi-level categorization addressing system (e.g., policies), organization (e.g., training opportunities), team (e.g., peer support and leadership), and individual (e.g., interface of individual practitioners and teams) level interventions. This evidence-informed toolkit was developed by the Canadian Health Workforce Network (CHWN), the Mental Health Commission of Canada (MHCC), and the University of Ottawa (uOttawa) as a part of Team Primary Care - Training for Transformation, an interprofessional initiative of the Foundation for Advancing Family Medicine. This initiative, funded by Employment and Social Development Canada (ESDC), is co-led by the College of Family Physicians of Canada and the CHWN. The aim of this initiative was to accelerate transformative change in the way primary care practitioners train to work by bringing together an extensive network of partners to increase the capacity of interprofessional comprehensive primary care through improved training for practitioners, supports for teams, and tools for planners and employers.

The toolkit supports healthcare trainees, workers, educators, managers, and leaders who collectively share a responsibility for creating and promoting a positive culture within healthcare teams and settings. Its overarching goal is to support comprehensive primary care teams and training programs to foster the PH&S of teams through the adoption and adaptation of a set of evidence-informed and sector-specific tools. Adopting a framework based on 7 themes, this article aims to describe the toolkit development process and how it addresses the key factors for psychologically healthy and safe healthcare workplaces.

## Methods

Our approach to a toolkit development was very comprehensive. We used a systematic environmental scan methodology targeting academic and grey literature focusing on 15 psychosocial factors to identify relevant resources that could support the PH&S of a diverse set of primary care practitioners in healthcare learning and working environments. To guide the process of resource selection for the toolkit, our team created a list of inclusion/exclusion criteria that specified date, language, intervention target and design, content/focus of intervention, setting,^1^ population, and country of origin of the resources we planned to collect. We aimed to include evidence-based resources used in different healthcare settings in Canadian and international contexts targeting different audiences (e.g., health workers, trainees, managers/supervisors/directors, healthcare educators/trainers, human resource representatives, and employees) at four different levels (system, organization, team, and individual)^2^ published in English or French between 2018 and 2023.

After the selection of resources for the toolkit had been made, we extracted them using an extraction tool that we created at the outset. In addition to basic information such as the resource’s name/title, hyperlink, and date, the extraction tool also captured other categories (i.e., theme/focus of intervention, country of origin, intervention audience, intervention level, cost, format, sector, healthcare setting, language, and populations) that served as a basis for the toolkit filters that were created subsequently.

To generate a guiding framework for the toolkit development, we started with the psychosocial factors that can affect PH&S in healthcare workplaces. Thirteen of these were identified in Canada’s National Standard for Psychological Health and Safety in the Workplace (The Standard),^1^ a set of voluntary guidelines designed to help organizations prevent psychological harm and promote psychological well-being which is recognized as a leading practice by the Health Standards Organization. Two additional factors (namely, protection from moral distress^3^ and support for psychological self-care) were identified by MHCC and HealthCare*CAN* as especially relevant to a healthcare environment.^10^ The reason for choosing these factors to form the basis of our framework is twofold. First, some evidence suggests that organizations implementing comprehensive, ongoing strategies addressing the PH&S of healthcare workers (such as the Standard) fare better than those that do not.^5,11^ Second, protection from moral distress and support for psychological self-care have been shown to be important factors to consider in relation to PH&S in healthcare settings.^10^

After a careful consideration of each psychosocial factor, aided by the team’s expertise assessing the relevance to team-based primary care and training, the team decided to cluster some of these together, given the synergies between them, resulting in 7 themes that guided the sorting of the resources in the toolkit: organizational and team culture, workload management and work-life balance, clear leadership and expectations, psychological protection, protection of physical safety, protection from moral distress, and support for psychological self-care (for a detailed description of each theme, please see Table 1). For instance, factors such as civility and respect, psychological and social supports, engagement, involvement and influence, and recognition and reward were included under one theme, namely, organizational and team culture. More details on the clustering of these different factors are included on the toolkit web site.

**Table 1.** Seven psychological health and safety themes and corresponding definitions.

Themes	Definition
Organizational and team culture	An organization’s culture is made up of the norms, values, beliefs, meanings, and expectations employees share and base their behaviour on.^1^ Positive traits of an organizational culture like trust, honesty, and fairness can promote psychological health and safety in the workplace.
Workload management and work-life balance	Having too much work and not enough time to do it is one of the most significant stressors in workplaces across Canada. To effectively balance work demands with their personal and family lives, primary care staff must have enough time to complete the tasks and responsibilities they are assigned.^1^
Clear leadership and expectations	Leaders provide clear, effective communication. They ensure that employees understand their roles and how they contribute to the organization. ^1^ Leaders provide helpful feedback on their expected and actual performance. Also, they ensure that employees are informed about important changes at work in a timely manner.
Psychological protection	A workplace is psychologically safe when staff feel comfortable enough to ask questions, seek feedback, report mistakes and problems, or propose a new idea. Leaders must also actively work to protect employees from stressors that could harm their mental health, including harassment, bullying, discrimination, violence, and stigma.^1,11^
Protection of physical safety	A physically unsafe environment creates concerns and anxiety among team members. For on-the-job safety, staff must be protected from hazards and risks in the physical work environment. Risks could stem from policies, training, a lack of demonstrated concern for employees’ physical safety, and other factors.^1^
Protection from moral distress	Staff should be able to perform their roles with a sense of integrity and the support of their profession, employer, and peers. Along with psychological self-care, this factor is especially critical in healthcare settings.^10^
Support for psychological self-care	Organizations and teams should encourage staff to care for their own psychological health and safety, including encouraging them to take breaks and vacations. Psychological self-care should be promoted and embedded into the organizational and team culture.^10^

On-line toolkit creation involved the design and development of an easy-to-navigate web site, the selection of 10 filters to quickly search and find suitable resources, and copyediting and translating English content into French to enhance accessibility. Web site creation was supported by Blue Eclipse, a professional Canadian web design company.

## Results

Guided by the framework underpinned by the 7 themes, we developed a toolkit that includes 122 resources. It comprises a multidimensional categorization addressing system, team, system, organization, and individual level interventions to enhance competencies of primary care practitioners to work more collaboratively within interprofessional team-based environments. It also integrates PH&S competencies into pre-licensure training programs. Examples of some resources related to specific themes are outlined in Table 2.

**Table 2.** Seven psychological health and safety intervention exemplars from the toolkit.

Themes	Exemplars
Organizational and team culture	Joy in work toolkit: This toolkit outlines essential components for fostering joy in the workplace and supporting the public health workforce. It also provides an action guide for leaders to promote staff well-being and cultivate joy at work.
Workload management and work-life balance	Time to speak Up: A graphical depiction of psychological safety in healthcare teams: An article that presents scenarios related to psychological safety in healthcare teams, while illustrating barriers and modelling a culture where individuals feel at ease to share their thoughts and concerns.
Clear leadership and expectations	Reflections on the mentor-mentee relationship: An article on how a successful mentoring relationship can bring significant benefits to mentees’ academic research productivity and career satisfaction. Also included is advice on how to choose a mentee or mentor, what each should bring to the relationship, and how the relationship can change over time.
Psychological protection	Mental health first aid: This is an evidence-based course on how to support a person who may be experiencing a decline in their mental well-being or a mental health crisis, until appropriate treatment is found, or the situation resolves. In addition to the 9-hour standard course, there are customized programs.
Protection of physical safety	Have THAT talk (for workplaces): A web page with a series of videos and worksheets on the 13 psychosocial factors defined in the National Standard of Canada for Psychological Health and Safety in the Workplace.
Protection from moral distress	Let’s talk moral distress: Resource guide: A resource to support teams who experience moral distress. It describes its underlying factors and offers strategies and videos to help workers and leaders take action to prevent and reduce moral distress.
Support for psychological self-care	Caring for healthcare workers: This resource features 2 tools: The Psychosocial Survey for Healthcare and the Organizational Review for Healthcare. Both can help healthcare organizations assess and promote psychological health and well-being in the workplace.

Our toolkit not only provides resources on specific components relevant to the PH&S of teams and trainees in primary care environments but also aims to educate users on key PH&S concepts. On the toolkit web page, under the “Home” tab, there are definitions of psychological health, psychological safety, and PH&S as well as definitions for each of the themes featured in the toolkit. The definitions of the concepts and themes are mostly based on definitions provided by organizations such as the Canadian Standard Association, the MHCC, and the Canadian Centre for Occupational Health and Safety.

A total of 87 resources in the toolkit focus on training/learning environments (40 for training/learning environments only and 47 apply to both training/learning and teams), while 81 resources target teams (35 for teams only and 47 applicable to both teams and training/learning environments).

Most of the resources (N = 92) were included under the team and organizational culture theme followed by psychological protection (N = 65), clear leadership and expectations (N = 23), workload management and work-life balance (N = 16), protection from physical safety (N = 15), and support for psychological self-care (N = 15). The theme with the least number of resources was protection from moral distress (N = 10).^4^

Given the process of clustering that underlies our framework, three of the themes (organizational and team culture, workload management and work-life balance, and psychological protection) covered in the toolkit have resources that address sub-themes (i.e., psychosocial factors/issues related to that specific theme). The resources included under the organizational and team culture theme address civility and respect, psychological and social supports, engagement, involvement and influence, and recognition and reward.^5^ Under the workload management and work-life balance theme, we included resources focusing on resource management and access, growth and development, psychological demands, and work-life balance. Resources included under the psychological protection theme address protection from bullying, harassment, violence, discrimination, and stigma related to mental health and/or substance use (self-stigma, interpersonal stigma, intersectional stigma, and structural stigma). Four of the themes (clear leadership and expectations, protection of physical safety, protection from moral distress, and support for psychological self-care) do not include sub-themes, so all resources included under these themes focus solely on the main thematic category.

Resources grouped under each of these different themes include both academic and non-academic literature such as articles, policies, guides, reports, training/workshops, podcasts, programs, surveys, webinars, videos, web sites, and other resource types. During the toolkit curation process, we aimed to ensure representation of various formats.

Given the importance of leadership in the creation and promotion of psychologically healthy and safe work and learning environments and workplaces, about half of the resources in our toolkit are intended for managers, supervisors and directors (N = 47), and human resource representatives (N = 17). The rest of the toolkit resources target health workers (N = 59), trainees (N = 35), employees (N = 3), and others (N = 4).

Under the “Resources” tab the toolkit has 10 filtering options to help users search and find the resources that best meet their interests and needs. Users can filter by theme, format, intervention level, audience, sector, setting, identity, cost, country, or language. The general search function can be utilized to type in a keyword or phrase such as “moral distress.” Users can also post a review after using a particular resource by leaving a comment and/or a star with a rating out of 5.

While our toolkit was launched on March 21, 2024, our web site data related to its usage to date seems promising. At the time of writing, close to 6,000 users have browsed the toolkit, out of which more than one thousand are based in Canada and the rest internationally.

## Discussion

Mindful of the need to devote greater attention to PH&S in the workplace in Canada and its importance in the quality of care in team-based primary care environments, we created a comprehensive evidence-informed toolkit to empower primary care teams and training programs to promote PH&S.

Recognizing the important role that leadership plays in advancing PH&S, we included many resources intended for managers, supervisors, and directors as well as human resource representatives. Many of the resources in the toolkit are also intended for trainees given their significant contributions in ensuring and promoting PH&S in primary care and other healthcare environments.

Developing an on-line, bilingual toolkit of resources for primary care teams and training programs is one part of a larger conversation needed to create collaborative, psychologically healthy and safe environments for workers and trainees. PH&S begins with rules/regulations within governments and professional institutions, continues with healthy work conditions created through policies, procedures, and activities by leadership and the organizations, and is sustained by the cumulative action of individual team members and trainees.^12^

Some of the key considerations emerging from the review of PH&S interventions are the importance of leadership prioritisation of interventions.^13^ An additional consideration is the level of awareness, willingness, and capacity to work on improving their PH&S.^5^ While organizational leaders and internal champions might be committed to this goal, starting a change initiative without assessing readiness may result in failure and compromise future efforts in cases when conditions are altered (e.g., introduction of new technologies).^5^

We hope the toolkit will significantly contribute to supporting primary care teams and training programs in fostering PH&S within interprofessional teams. To that end, we will continue mobilizing it through partners’ networks, conferences, and partnerships with key stakeholders. Indeed, the implementation of the interventions in the toolkit is the key next step for which health system leadership is critical.

## Limitations

While our toolkit incorporates many Canadian and internationally based resources in a variety of formats, its inventory is by no means exhaustive. Indeed, our search process for the toolkit was finalized in July 2023. New resources might have emerged in the meantime, and we will strive to include them as well as to ensure that resource links are updated regularly. Moreover, our review of the literature revealed some gaps in terms of available resources which may have limited the choice of the resources included in the toolkit.

First, there is a relative lack of bilingual resources and resources published in French compared to English publications. Also, many resources have not focused on primary care settings. Interestingly, few resources focus on interprofessional teamwork or on preparing future professionals for interprofessional collaboration. Most importantly, team and training resources targeting individuals are more prominent than those targeting the team or system levels, but few focus on the interface of individuals within teams. Lastly, system-level interventions do not focus on the primary care team and training relevant circumstances. Finally, the team did not assess the quality of included resources; it simply deemed them evidence-informed promising practices worthy of consideration. Consequently, their inclusion in this toolkit should not be considered an endorsement of particular resources and/or organizations.

The relative lack of system and organizational level interventions is concerning given that evidence shows these interventions can be more effective than individual level interventions in alleviating work-related mental health, stress, and burnout.^14^ In fact, research has shown that a multi-faceted approach to interventions is most effective and thus, individual level interventions should be understood to be complementary to organization and system level approaches.^14,15^

Lastly, while we did not have an opportunity to evaluate the toolkit via health leader interaction with it given its very recent launch, it is our intention to do so soon. We hope to receive feedback from leaders in primary care environments which would not only allow us to strengthen the toolkit but also indicate the way in which similar toolkits could be improved.

## Conclusion

The evidence-informed toolkit we created to empower primary care teams and training programs is an important step toward creating and promoting psychologically healthy and safe healthcare workplaces and learning environments in primary care contexts. Commitment is needed from all primary care team members—leaders, educators, human resources representatives, and health workers toward the same goal of building PH&S into workplaces. Readiness of organizations and training programs to adopt the resources and implement changes is critical. In addition to commitment and organizational readiness, more resources must be developed, specifically those focusing on gaps identified through our literature review. In particular, we need more resources that focus on primary care settings, interprofessional teamwork or preparation of future professionals for interprofessional collaboration, and are team or system level. We need more system-level interventions/resources that focus on the primary care team and training relevant circumstances.

## Notes

1. The settings included learning environment, community care, home care, on-line setting, hospital, long-term care, private clinic, and general and other healthcare setting.

2. Individual level (only if targeting those in leadership or supervisory positions; very specific to context of healthcare, focus on empowerment and changes in work culture). Team level (e.g., peer support and mentorship). Organization level (e.g., policies and leadership positions). System level (e.g., policies and collective agreements).

3. Moral distress happens “when one knows the ethically correct action to take but feels powerless to take that action” due to internal or external constraints.^16^

4. Please note that many resources in the toolkit focus on more than one topic and are geared toward multiple audiences.

5. For definitions of specific themes and sub-themes, please refer to our toolkit web site.
